# Prediction of High Incidence of Dengue in the Philippines

**DOI:** 10.1371/journal.pntd.0002771

**Published:** 2014-04-10

**Authors:** Anna L. Buczak, Benjamin Baugher, Steven M. Babin, Liane C. Ramac-Thomas, Erhan Guven, Yevgeniy Elbert, Phillip T. Koshute, John Mark S. Velasco, Vito G. Roque, Enrique A. Tayag, In-Kyu Yoon, Sheri H. Lewis

**Affiliations:** 1 Johns Hopkins University Applied Physics Laboratory, Laurel, Maryland, United States of America; 2 Department of Virology, Armed Forces Research Institute of Medical Sciences, Bangkok, Thailand; 3 National Epidemiology Center, Department of Health, Manila, Philippines; Oswaldo Cruz Foundation, Brazil

## Abstract

**Background:**

Accurate prediction of dengue incidence levels weeks in advance of an outbreak may reduce the morbidity and mortality associated with this neglected disease. Therefore, models were developed to predict high and low dengue incidence in order to provide timely forewarnings in the Philippines.

**Methods:**

Model inputs were chosen based on studies indicating variables that may impact dengue incidence. The method first uses Fuzzy Association Rule Mining techniques to extract association rules from these historical epidemiological, environmental, and socio-economic data, as well as climate data indicating future weather patterns. Selection criteria were used to choose a subset of these rules for a classifier, thereby generating a Prediction Model. The models predicted high or low incidence of dengue in a Philippines province four weeks in advance. The threshold between *high* and *low* was determined relative to historical incidence data.

**Principal Findings:**

Model accuracy is described by Positive Predictive Value (PPV), Negative Predictive Value (NPV), Sensitivity, and Specificity computed on test data not previously used to develop the model. Selecting a model using the F_0.5_ measure, which gives PPV more importance than Sensitivity, gave these results: PPV = 0.780, NPV = 0.938, Sensitivity = 0.547, Specificity = 0.978. Using the F_3_ measure, which gives Sensitivity more importance than PPV, the selected model had PPV = 0.778, NPV = 0.948, Sensitivity = 0.627, Specificity = 0.974. The decision as to which model has greater utility depends on how the predictions will be used in a particular situation.

**Conclusions:**

This method builds prediction models for future dengue incidence in the Philippines and is capable of being modified for use in different situations; for diseases other than dengue; and for regions beyond the Philippines. The Philippines dengue prediction models predicted high or low incidence of dengue four weeks in advance of an outbreak with high accuracy, as measured by PPV, NPV, Sensitivity, and Specificity.

## Author Summary

A largely automated methodology is described for creating models that use past and recent data to predict dengue incidence levels several weeks in advance for a specific time period and a geographic region that can be sub-national. The input data include historical and recent dengue incidence, socioeconomic factors, and remotely sensed variables related to weather, climate, and the environment. Among the climate variables are those known to indicate future weather patterns that may or may not be seasonal. The final prediction models adhere to these principles: 1) the data used must be available at the time the prediction is made (avoiding pitfalls made by studies that use recent data that, in actual practice, would not be available until after the date the prediction was made); and 2) the models are tested on data not used in their development (thereby avoiding overly optimistic measures of accuracy of the prediction). Local public health preferences for low numbers of false positives and negatives are taken into account. These models appear to be robust even when applied to nearby geographic regions that were not used in model development. The method may be applied to other vector borne and environmentally affected diseases.

## Introduction

Dengue fever is a common human viral disease transmitted via the bite of infected *Aedes* mosquitoes, typically *Aedes aegypti*. These mosquitoes are capable of breeding in uncovered containers holding rain water, such as tires, buckets, flower pots, etc., that are commonly found in urban areas in the tropics [1]. Dengue incidence has increased 30-fold over the last 50 years, is endemic in more than 100 countries, and causes an estimated 50 million infections annually [2]. Dengue has been cited as the most important arthropod-borne viral disease of humans, with an estimated 2.5 billion people globally at risk [3]. Bhatt et al. [4] recently used a cartographic approach to estimate that there may be as many as 390 million dengue infections annually, which is more than three times the global dengue burden estimated by the World Health Organization (WHO).

Dengue has a wide clinical spectrum ranging from asymptomatic to severe clinical manifestations [2]. The classic presentation (called dengue fever or DF) begins with an abrupt onset of high fever, often accompanied by erythema, severe muscle and joint pain, headache, nausea, and vomiting [5]. Recovery is prolonged and marked by fatigue and depression [6]. There are four known serotypes of the virus, although the initial clinical presentations are almost identical [3]. A severe presentation, known as dengue hemorrhagic fever (DHF) occurs primarily in patients who are re-infected with a different serotype [7]. DHF includes increased capillary permeability with potentially significant vascular leakage that compromises organ function and may lead to shock [2]–[3], [5]–[7]. Mortality in DHF has been reported as high as 10–20% and over 40% if shock occurs [8]. Prevention methods currently rely on vector control until such time as a specific anti-viral therapy or a licensed vaccine becomes available [9]. Therefore, public health agencies see potential benefit if a means could be found to predict dengue outbreaks with enough advance time to allow for the planning and implementation of mitigation strategies. An effective prediction model would be particularly helpful in areas where resources for such efforts are limited and in locations where medical treatment facilities may become overwhelmed by an outbreak.

Many published studies have described the association of different parameters with dengue outbreaks. Most of these studies have examined the environmental factors that influence mosquito vector populations [e.g.], [ 1], [10,11]. While some studies used only simulated data to develop methods for capturing seasonal forcing [e.g., 12], others used real data. In 2008, Runge-Ranzinger et al. [13] performed a systematic literature review in order to identify dengue outbreak prediction models or detection methods. One outcome of this literature review was the observation that most studies were retrospective and primarily focused on dengue outbreak detection and surveillance, rather than prediction. Furthermore, it was determined that most studies lacked an appropriate determination of sensitivity and specificity. A more recent literature review of decision support systems for the prediction, prevention, and control of vector-borne diseases was carried out in 2011 by Eisen and Eisen [14]. While the focus of this literature review was on geographic information systems and remote sensing data, it was determined that the studies reviewed provided useful risk maps, but did not provide actual predictions or forewarnings of future outbreaks. Finally, in addition to the studies evaluated in these two literature reviews, there are a variety of published methods for the surveillance and detection of an outbreak that has just begun but is not yet obvious [e.g., 15].

However, there seem to be fewer methods described in the literature for predicting a dengue outbreak well before it has begun (i.e., prediction). Among the latter is a study by Yu et al. [16] that analyzed climate data (including temperature, rainfall, and Southern Oscillation Index), mosquito larvae abundance, and human health data to develop a Bayesian Maximum Entropy model to predict dengue outbreaks one week in advance. Unlike most studies, Yu et al. developed their model on one set of data but tested it on a different set of data, thereby avoiding the exaggerated performance metrics that may occur when the same dataset is used for both development and testing. They found that the probabilities that a dengue outbreak actually occurred given a positive prediction and given a negative prediction were 0.5541 and 0.031, respectively. A study by Hii et al. [17] described a time series Poisson multivariate regression model to predict weekly dengue cases in Singapore using temperature, rainfall, and previous dengue incidence. After developing this model using 2000–2010 dengue data, it was used to predict dengue incidence during the first six weeks of 2011. They then used observed weather data and the previously predicted dengue incidences for prediction of dengue incidence during weeks 7–16 of 2011. A recent study by Lowe et al. [18] described using a Bayesian generalized linear mixed model approach to produce probabilistic predictions of monthly dengue incidence in Brazil. It is noteworthy that, like the present study, these authors used different data for development and testing, thereby allowing for more realistic accuracy assessments.

Some recent studies have applied data mining techniques to human health and environmental data to support the predictive modeling of dengue outbreaks [19], [20]. Traditional regression-based methods may be difficult to use with nonlinear models and high dimensional data containing many potentially interacting predictor variables [21]. Data mining techniques are particularly appropriate for large datasets with variables that may interact in complicated ways. These techniques automatically search for relationships that may include potentially large numbers of main effects and complex interactions. Only the association rules whose metrics meet the pre-determined criteria of significance are selected, thus ensuring that variables that are not strongly correlated are excluded from the final model.

As an example of a data mining/machine learning technique, Bakar et al. [19] developed five types of classifiers for predicting dengue outbreaks: Decision Tree, Rough Set Classifier, Naïve Bayes, Association Rules-based classifier, and a multiple classifier that combined results from each of the aforementioned classifiers. Medical records of dengue patients from the Selangor area in Malaysia served as the data source for this approach. Their classifiers did not predict area outbreaks for dengue but instead predicted whether or not a patient was a repeat dengue case. Finally, Buczak et al. [20] developed data mining techniques for the prediction of multi-week dengue incidence in Peru four to seven weeks in advance. The objective of the present study is to describe further development of these techniques and their application to the prediction of weekly dengue incidence in specific Philippines provinces four weeks in advance.

## Materials and Methods

The prediction method uses types of data selected from studies in the literature that have shown significant correlation with dengue incidence. These data include previous dengue incidence data, remotely sensed meteorological data (i.e., land surface temperature, rainfall), remotely sensed vegetation data, climate data (sea surface temperature anomalies, Southern Oscillation Index), and socio-economic data (population, sanitation). The data sources for the different variables are shown in Table 1. It should be noted that complex mechanisms and interactions might lead these variables to be significant indicators of future dengue activity.

**Table 1:** Data sources.

Data type	Source
**Rainfall**	NASA Tropical Rainfall Measuring Mission http://mirador.gsfc.nasa.gov
**Temperature**	USGS Land Processes Distributed Active Archive Center https://lpdaac.usgs.gov/get_data
**Typhoon Status and Wind**	Unisys Weather http://weather.unisys.com/hurricane/w_pacific
**NDVI**	USGS Land Processes Distributed Active Archive Center https://lpdaac.usgs.gov/get_data
**EVI**	USGS Land Processes Distributed Active Archive Center https://lpdaac.usgs.gov/get_data
**Southern Oscillation Index**	US National Center for Atmospheric Research http://mirador.gsfc.nasa.gov
**Sea Surf. Temp. Anomaly**	NASA Global Change Mastery Directory https://lpdaac.usgs.gov/get_data
**Altitude**	NOAA National Geophysical Data Center http://www.ngdc.noaa.gov/mgg/topo
**Socio-economic**	Philippines National Statistics Office http://census.gov.ph
**Political Stability**	Worldwide Governance Indicators Project http://info.worldbank.org/governance/wgi/index.asp

### Epidemiological and Socioeconomic Predictor Data

The mosquito vector acquires the dengue virus from biting a human and maintains disease endemicity by passing it to another human [1]. Therefore, previous human incidence rates reflect the presence of the virus in the local human population. The presence of the virus may also be assessed by the determining the number of infected mosquitoes, but these data are time-consuming and expensive to obtain for a large geographic area such as Philippines [1] and we did not have access to such data. Socio-economic conditions may also contribute to human exposure to the mosquito vector [22].

#### Data collection

The socioeconomic data of Philippines included poverty index, electricity access, drinking water access, sanitation index, and child development indices (child health, child education, quality of life), and these data were downloaded from the Philippines National Statistics Office website [23]. While these data have different geographical resolutions, they have been mapped to the resolution of a Philippine province (see Standardization of Geographic Information section below). Data for missing years are linearly interpolated from the existing years and extrapolated for the current year.

The epidemiological data were provided by the National Epidemiology Center of the Republic of the Philippines (RP) Department of Health. Before 2008, dengue data were collected under the National Epidemic Sentinel Surveillance System (NESSS), which used data from multiple hospital surveillance sites. Following the 2002 emergence of Severe Acute Respiratory Syndrome (SARS), the Asian Development Bank funded a Regional Technical Assistance for Strengthening Epidemiologic Surveillance and Response (ESR) for Communicable Diseases in Indonesia, Malaysia, and the Philippines. NESSS and other disease surveillance systems in the RP were subsequently merged into PIDSR (Philippines Integrated Disease Surveillance and Response system), which became operational in 2008 and 2009. Some of the data collection, processing tools, and methods were changed under this new system, resulting in increased reporting of suspected dengue visits and the collection of more complete information. Available health data thus represented suspected dengue hospital visits from NESSS and PIDSR from the beginning of 1993 to the end of 2011. The NESSS data were primarily provided in an Epi Info [24] format; however, all data since 2003 were packaged in Microsoft (MS) Access tables.

#### Data validation and processing

**Figure 1:**
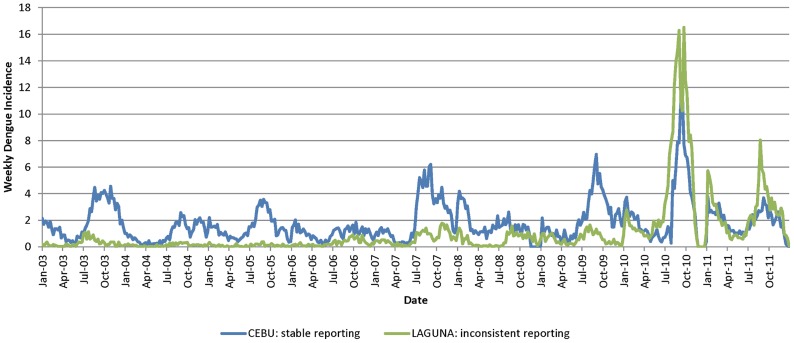
Weekly incidence for Cebu and Laguna provinces.

**Figure 2:**
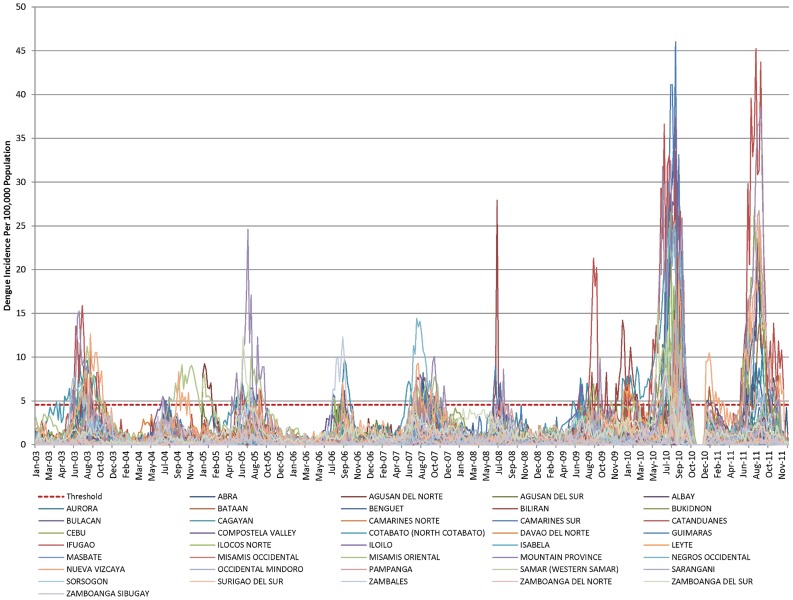
Philippines dengue incidence per province.

SAS (Statistical Analysis Software) version 9.3 [25] was used for the pre-processing of epidemiological data. Data from different years were combined using a set of common variables from relevant fields that were consistently filled: Region, Province, Municipality, Barangay, Address, Outcome, Sex, Age, Hospital name, and Case classification. To take into account the transition from NESSS to PIDSR around 2008, the dengue reporting data was evaluated for consistency over the full range of years. The data from 1993 through 2002 were deemed to be too dissimilar from the later years in terms of fields populated and reporting characteristics and were thus excluded from the study. A data quality check and discussion with the RP data providers revealed some additional reporting problems in later years. The most significant was a reporting gap starting around the middle of October to the end of December in 2010 (noticeable on Figures 1 and 2). For that reason, data from 10/17/2010 to 3/21/2011 were excluded, with the additional weeks allowing for a buffer period to absorb the effect of bad reporting. Except for this exclusion, data from 2003 through 2011 were used in this study because these data had similar fields and reporting characteristics.

#### Standardization of geographic information

The geographic divisions of the Philippines consist of 17 regions divided into 80 provinces and the Manila Metropolitan Area. The provinces consist of roughly 1,650 cities and municipalities, which in turn comprise about 42,000 barangays (i.e. neighborhoods). The numbers of municipalities and barangays fluctuate from year-to-year. Examination of the geographic information contained in the dengue data revealed that smaller political subdivisions (i.e. finer spatial resolution) have a greater percentage of missing geographic data (see Table 2). To fill some of these missing data, spatial information from the dengue data was matched to freely available data from the Philippines Geographic Information System (GIS) Data Clearinghouse [26].

**Table 2:** Matched geographic information between dengue visits data and GIS file.

Geography	Total #	Missing	Match (after standardization)
**Region**	17	0%	100%
**Province**	80	1%	99%
**Municipality**	1,650	3%	96%
**Barangay**	42,000	30%	35% (No matching done)

Province information was missing for approximately 1% of the dengue data. After the pre-processing step, which includes the removal and replacement of special characters and capitalization, approximately 200 mismatches remained among the values in the province field. Some of these mismatches were due to abbreviations, typos, lower case letters, and outdated information. All of the mismatches had an obvious designation in the GIS file. For example, “Davao, North” was “Davao Del Norte” and “Western Samar” was “Samar.” Therefore, the province name was replaced with the corresponding spelling used in the GIS file. Some of the missing province information was imputed from the available municipality geographic information.

Municipality information was missing about 3% of the time, and about 4000 of the municipality values also differed from those in the GIS municipality table after pre-processing. As was done in the case of provinces, each mismatch was examined separately. After the municipality name was replaced with the one designated for it in the GIS table, the match rate with GIS data improved to 96%.

A summary of the results of matching patient address location at different spatial resolutions to the GIS data file is presented in Table 2. The decision was made to use the province as the basic spatial unit for analysis because province data contained a greater proportion of matched data and a better density of visits over the study period than smaller political divisions (e.g., barangay). In order to estimate incidence rates for each of the years from 2003 to 2011, the denominators were derived from a linear fit to values for province populations from the official RP census for 1995, 2000, and 2007. Finally, daily dengue incidence data were pre-processed into weekly data in order to mitigate issues, such as missing data and day-of-week effects, and to increase compatibility with weekly environmental data.

#### Selection of provinces for analysis

For each RP province, weekly dengue incidence rates were determined and plotted as a time series in order to assess whether the data were stable enough to be used for modeling. Analysis of weekly incidence rates of province-level data revealed inconsistencies and frequent changes in reporting. For example, the weekly dengue incidence for the provinces of Laguna and Cebu are plotted on Figure 1. While the incidence rate is relatively stable throughout the analysis period for the Cebu province, there is a significant trend for Laguna province resulting from increased reporting in the later years. For all the RP provinces, we identified those that had significant reporting trend (often due to the fact that additional hospitals started reporting), reporting variability, data drop-offs, and inconsistencies from the time series plots. Unfortunately, there were no province-level data for confirmed dengue incidence to make reliable distinction between reporting trend and elevation of disease incidence. As a result of this analysis, it was determined that 40 out of 81 provinces had relatively robust reporting. Table 3 shows the resulting provinces that were selected for further analysis.

**Table 3:** Provinces selected for further analysis.

Selected	Not Selected
Abra	Aklan
Agusan del Norte	Antique
Agusan del Sur	Apayao
Albay	Basilan
Aurora	Batanes
Bataan	Batangas
Benguet	Bohol
Biliran	Camiguin
Bukidnon	Capiz
Bulacan	Cavite
Cagayan	Davao del Sur
Camarines Norte	Davao Oriental
Camarines Sur	Dinagat Islands
Catanduanes	Eastern Samar
Cebu	Ilocos Sur
Compostela Valley	Kalinga
Cotabato	La Union
Davao del Norte	Laguna
Guimaras	Lanao del Norte
Ifugao	Lanao del Sur
Ilocos Norte	Maguindanao
Iloilo	Marinduque
Isabela	Metro Manila
Leyte	Negros Oriental
Masbate	Northern Samar
Misamis Occidental	Nueva Ecija
Misamis Oriental	Oriental Mindoro
Mountain Province	Palawan
Negros Occidental	Pangasinan
Nueva Vizcaya	Quezon
Occidental Mindoro	Quirino
Pampanga	Rizal
Samar	Romblon
Sarangani	Siquijor
Sorsogon	South Cotabato
Surigao del Sur	Southern Leyte
Zambales	Sultan Kudarat
Zamboanga del Norte	Sulu
Zamboanga del Sur	Surigao del Norte
Zamboanga Sibugay	Tarlac
	Tawi-Tawi

#### Threshold determination

The threshold between ***Low*** and ***High*** incidence needs to be determined before the rules are extracted from the data and the classifiers performing predictions are built. While the threshold could be set based upon an outbreak definition, several different outbreak definitions exist, depending upon the context and the disease [27], [28], and are often determined by such factors as expert advice or assuming the incidence is Gaussian. Generally, a threshold should be set high enough to reduce the chances of statistical and non-outbreak fluctuations in incidence data causing outbreak alarms. If the threshold is set too low, situations of “alarm fatigue” may occur [29]. In resource-limited settings, having a low false alarm rate helps to conserve those resources for situations in which the accurate prediction of an outbreak is more likely. Therefore, the threshold needs to be meaningful to the user and linked to the action to be taken when the predicted value exceeds the threshold.

The threshold between ***Low*** and ***High*** incidence was determined based on historical data and on feedback from local public health professionals regarding what is operationally meaningful. As described in Lowe et al. [18], it is important that such thresholds are carefully designed to minimize false alarms and false negatives and to reflect the response capabilities of the local public health professionals. Figure 2 shows the historical incidence for the 40 selected provinces for the period January 2003 through December 2011. The historical mean and standard deviation of the incidence data were computed for the period January 2003 through December 2011. Based upon user feedback, the threshold between ***Low*** and ***High*** was set at the mean plus 1.5 standard deviations, which corresponds to a weekly incidence of 4.554 per 100,000 population.

#### Use of dengue data from adjacent provinces

In addition to the past dengue incidence data described above, four derived predictor variables describing dengue incidence in adjacent provinces were calculated. Each province was defined by its shape in the Philippines GIS database and adjacent provinces were identified by running a Structured Query Language (SQL) statement on this GIS database. Then, for each province, the dengue incidence rates in its neighbors were aggregated to form four predictor variables: *mean*, *maximum*, *median*, and *overall adjacent incidence*. The mean, maximum, and median adjacent incidence rates were defined, respectively, as the mean, highest, and median values of dengue incidence of all the neighboring provinces combined. The *overall adjacent dengue incidence* was computed as the sum of dengue case counts in the neighboring provinces divided by the sum of populations of those provinces.

### Environmental Predictor Data

Abnormally high or low temperatures may reduce mosquito longevity and reproduction because of their effect on biological parameters such as the extrinsic incubation period of the mosquito [1], [10]. Eight-day mean land surface temperature data were obtained from the United States Geological Survey (USGS) Land Processes Distributed Active Archive Center (LPDAAC), which provides data remotely sensed by the Moderate Resolution Imaging Spectroradiometer (MODIS) instrument onboard two US satellites [30]. Land elevation also results in variations in seasonal temperatures. Therefore, land elevation data were obtained from the US National Oceanic and Atmospheric Administration National Geophysical Data Center (NGDC) [31].

For the completion of its life cycle, the mosquito vector requires breeding sites, such as stagnant water in containers and natural pools, which can be filled by rainfall. Heavy rainfall may wash the larvae away, whereas too little rainfall may limit the breeding sites [1]. Rainfall rate data derived from measurements made by the NASA Tropical Rainfall Measuring Mission (TRMM) satellites were obtained from the NASA Goddard Earth Sciences Data and Information Services Center [32]. Typhoons, which frequently pass nearby or make landfall in the Philippines, bring heavy rainfall and high winds that may result in infrastructure damage. After storm passage, infrastructure damage and remaining stagnant pools of water may lead to increased exposure of the human population to the mosquito vector. Therefore, typhoon intensity and associated wind data were obtained from the US Naval Oceanographic Command Joint Typhoon Warning Center (JWTC) Best Track data [33]. These data consist of the entire typhoon path coordinates and times, and the maximum sustained wind speed at each storm location. The time resolution of the storm position was six hours. Using a radius of 80 km around each storm path coordinate, it was determined whether the interior of this circle intersected a Philippines province. If the intersection of this circle with land for a province was a non-empty set, the typhoon status variable was assigned a value according to the US Naval Oceanographic Command Joint Typhoon Warning Center typhoon categorization (dissipated, tropical depression, tropical storm, typhoon-1, typhoon-2, typhoon-3, typhoon-4, and typhoon-5) and the maximum sustained wind data were included in the predictor variable data. Otherwise, the typhoon status and maximum sustained wind variables were set to null values.

Vegetation type and biomass may be indicative of the amount of moisture in the soil and may also reflect human activity such as deforestation. Satellite measurements of vegetation indices have been used to assess green leaf biomass, photosynthetic activity, and the effects of seasonal rainfall, which are then related to vector habitat characteristics and disease outbreaks [34]. Sixteen-day mean vegetation index data, which are measured by the MODIS instrument, were obtained from the United States Geological Survey (USGS) Land Processes Distributed Active Archive Center (LPDAAC) [30]. These vegetation index data include the Normalized Difference Vegetation Index (NDVI) and the Enhanced Vegetation Index (EVI). NDVI is closely related to photosynthetic activity, vegetation type, and landscape disturbance [35]. EVI is similar to NDVI but corrects for atmospheric effects, is more sensitive to variations in canopy structure, and provides for a higher resolution vegetation index scale in regions of dense biomass [36].

The Southern Oscillation Index (SOI) is a single global number derived from the atmospheric pressure difference between Darwin, Australia, and Tahiti, French Polynesia. As wind blows from high to low pressure, the strength of the pressure difference influences the strength of the wind and the wind-driven movement of the surface water. These conditions influence both regional and global weather patterns. The sign of the SOI determines whether conditions are neutral, El Niño, or La Niña, while the magnitude of the SOI indicates the strength of the El Niño and La Niña. El Niño and La Niña conditions typically result in rainfall anomalies (e.g., heavy rain, drought) and temperature anomalies a few months later in regions both nearby and some considerable distance from the tropical Pacific [37]. Sea Surface Temperature Anomaly (SSTA) data are also used as more local indicators of near-term future rainfall anomalies [38]. Higher sea surface temperatures (e.g., warm SSTA) result in more convection and the creation of rain-forming clouds. These clouds will be blown by the winds either away from or toward land, resulting in changes in rainfall patterns that may not be seasonal. Therefore, the SOI and SSTA data are used in order to provide leading indicators of future weather patterns that may not always be seasonal. Monthly SOI [39] and weekly SSTA data [40] are reported by the US NOAA National Weather Service Climate Prediction Center.

The data described above were pre-processed to fit a single spatiotemporal resolution: one week and one Philippine province (political divisions of the Philippines will be described below under Standardization of Geographic Information). This pre-processing involved aggregation and/or interpolation of data into weekly values for each province in the Philippines, the details of which are found in Buczak et al. [20].

### Prediction Methodology

**Figure 3:**
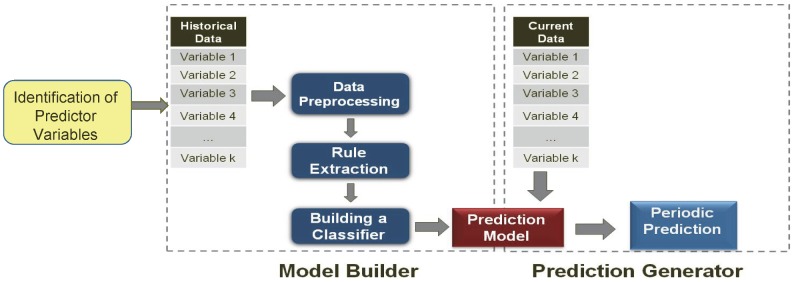
Dengue prediction method.

The prediction method performs data mining from a large number of data sources, following the steps shown in Figure 3. The three main steps are summarized here and described below.

1. Identification of Predictor Variables
2. Model Builder
  - Data pre-processing and fuzzification
  - Rule extraction using Fuzzy Association Rule Mining
  - Rule selection using specific metrics
3. Prediction Generator

1. **Identification of the Predictor Variables:** The identification of the predictor variables is a manual process in which a literature review is performed to identify the environmental and socio-economic variables that are correlated with the given disease incidence. After the identification of data sources for predictor variables, data are downloaded. The variables used for dengue prediction were described in the previous sections.
2. **Model Builder:** The Model Builder is the principal part of the method and is where all the data mining elements reside. The data are pre-processed and used to find fuzzy association rules. A subset of these rules that satisfy certain criteria is then selected to create a classifier that becomes the Prediction Model.
  a) Data pre-processing and fuzzification: The predictor variable data are pre-processed to convert them into the desired spatio-temporal resolution, as described in detail in Buczak et al. [20]. For the Philippines, the spatial resolution is one province and the temporal resolution is one week.

**Figure 4:**
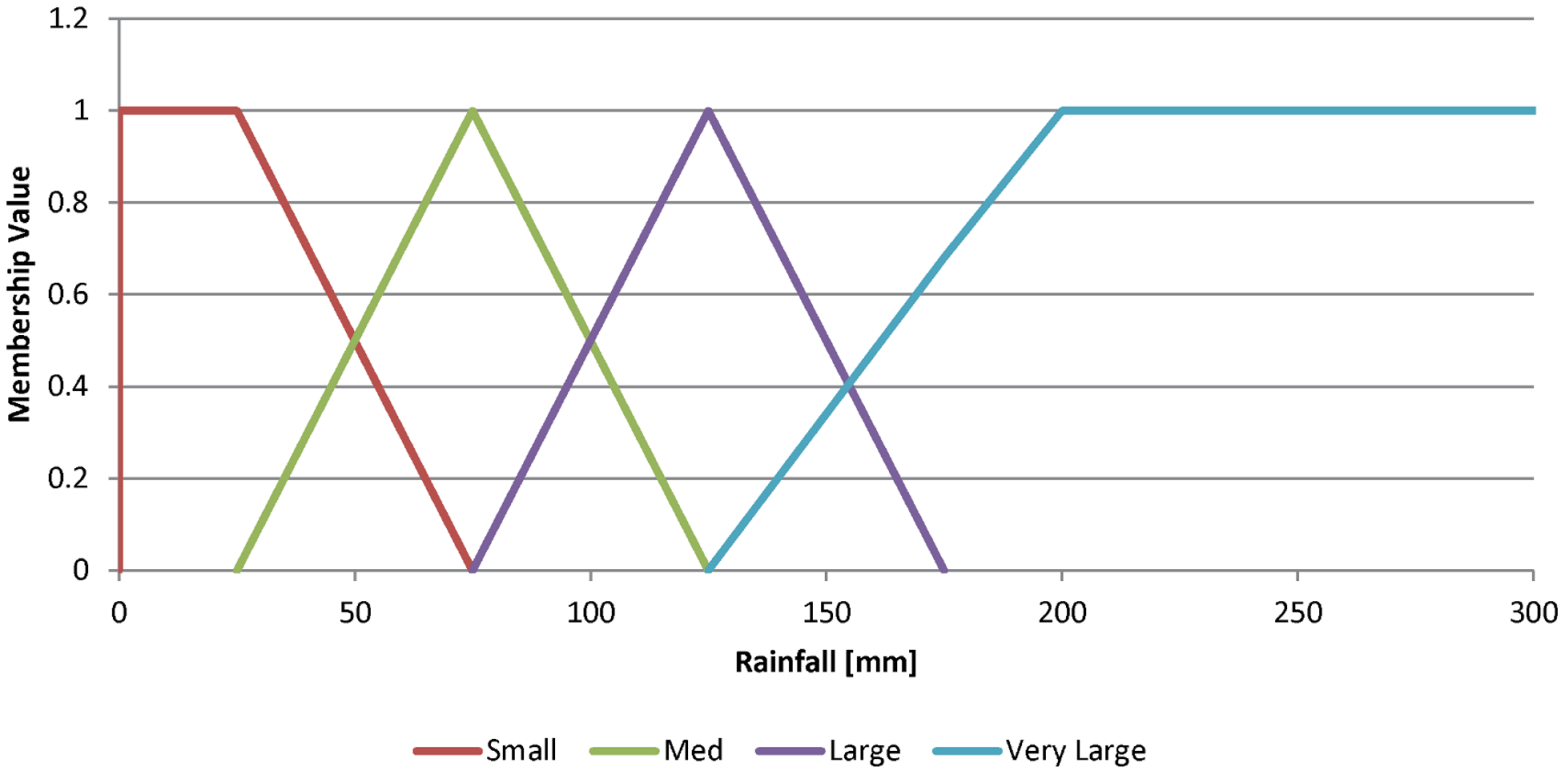
Membership functions for the variable Rainfall: *SMALL*, *MED*, *LARGE*, *VERY LARGE*.

The data are then transformed into membership values (i.e., fuzzified) using fuzzy set theory [41]. Fuzzy membership functions are defined for each variable. As an example, four fuzzy membership functions (***SMALL***, ***MED***, ***LARGE***, ***VERY LARGE***) for the variable *Rainfall* are shown in Figure 4. Fuzzification is defined as the process in which a number (e.g. rainfall value in mm) is transformed into a membership value lying between 0 and 1, thereby allowing for a smooth transition between full membership (1) and non-membership (0). The degree of membership in a set is generally considered to be the extent to which a corresponding fuzzy set applies. In Figure 4, a *Rainfall* of 50 mm will be transformed into two membership functions ***SMALL*** with a degree of membership 0.5 and ***MED***, with a degree of membership 0.5.

  b) Rule extraction: Rule extraction from the training data is the most important part of the entire method. It is performed using Fuzzy Association Rule Mining (FARM) [42], a set of data mining methods that use a fuzzy extension of the Apriori algorithm [43] to automatically extract from data the so-called *fuzzy association rules*. Fuzzy association rules are of the form:
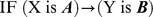
where X and Y are variables, and ***A*** and ***B*** are membership functions that characterize X and Y respectively. X is called an antecedent and Y is called a consequent of the fuzzy association rule. An example of a fuzzy association rule (not used in dengue prediction) is:
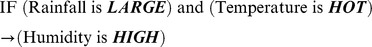

This rule uses the linguistic term (fuzzy set) ***HOT*** for temperature, wherein temperatures of 70F, 80F, and 100F could each be considered to have a degree of membership of 0.1, 0.8, and 1, respectively, in the fuzzy set ***HOT***. An important advantage of the fuzzy association rules is that they are easily understood by humans because of the linguistic terms that they employ (e.g., ***LARGE***, ***HOT***, ***HIGH***).For the disease prediction application, the rules of interest are called *fuzzy class association rules* (FCARs), meaning that they have only one consequent: the class. An example of a FCAR extracted by FARM is:
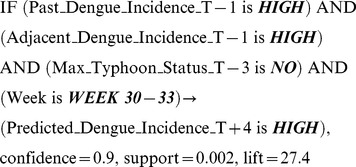
The above rules states that if Dengue Incidence one week ago (T-1) was ***HIGH*** and Adjacent Dengue Incidence one week ago (T-1) was ***HIGH*** and Typhoon Status three weeks ago (T-3) was ***NO*** and the week for which the prediction is made is between 30 and 33 (these numbers refer to US CDC epidemiological weeks), then the Dengue incidence will be ***HIGH*** 4 weeks from now (T+4). The terms confidence, support, and lift are metrics used in the rule selection to be described next.

  c) Rule selection: In order to build the classifier that will perform the final prediction, it is necessary to determine which rules, of the thousands extracted by FARM, should be used and in which sequence to use them. An automatic method is used to choose a small subset of rules that minimizes the misclassification error on the fine-tuning data set. The rule choices are based on selection criteria using the three most important metrics for fuzzy association rules: confidence, lift, and support.

*Confidence* can be considered to be the conditional probability that, if the antecedents are true, then the consequent is true. A rule with confidence of 1 is always true. *Support* is a measure of how general a given rule is. Support can be considered to be the probability of occurrence of records with given antecedents and consequent in a particular data set. A support of 0.01 means that a given rule describes 1% of a particular data set. *Lift* represents the extent to which the antecedents and the consequents are not independent. The higher the lift, the more dependent the variables are. A thorough description of the rule metrics and associated equations can be found in [43], [20].The method for building the classifier is based on extensions of the method of Liu et al. [44], as described in Buczak et al. [20]. The following extensions were developed:

  - Order all the rules first by confidence, next by lift, and finally by the number of antecedents.
  - Weight the misclassification error. The user has the opportunity to give a much higher weight for misclassifying the cases with a small number of exemplars than with a large number of exemplars. This is related to the fact that, in the datasets used, about 96% of the data were from class ***LOW***.

The classifier is an ordered set of rules. Once new data arrive and are preprocessed, the highest ranked rule in the classifier that matches the antecedent of the rule is executed. The consequent of that single rule constitutes the prediction for the given data point. The last rule in the classifier, in addition to antecedents and consequent, has also a *default class*. The default class is invoked only when the input data do not fit the antecedents of any rule in the classifier. In this case, if the default class is ***LOW***, then the prediction is ***LOW***; if the default class is ***HIGH***, the prediction is ***HIGH***. The final classifier selected based on the criteria described above constitutes the Prediction Model.

3) **Prediction Generator:** The Prediction Generator is a straightforward process that periodically computes predictions using the Prediction Model built in Step 2. Because the temporal resolution is one week, a new prediction can be computed weekly when new input data are available. It is important to emphasize that this model only uses data that would actually be available on the date the prediction was generated. For example, when doing a prediction during week T if some weekly data for week T-1 were typically not available (e.g. temperature data is only available for week T-2 because of latency of with which that data can be accessed), then that data would not be used as input to this model. Once available, these new data automatically undergo pre-processing and are fed into the Prediction Model that computes predictions, which are then displayed on a map. The outcome variable (predicted dengue incidence) is converted to a binary variable, either ***HIGH*** or ***LOW*** dengue incidence (where the threshold between high and low values was quantitatively defined as the historical mean dengue incidence plus two standard deviations).

### Classifier and Rule Generation Improvements

For the present study, enhancements were made to the classifier building algorithm described in Buczak et al. [20]. The first enhancement was to automate the process of finding optimal misclassification weights. As described in [20], user-specified weights on the misclassification errors were employed in order to place a higher penalty on misclassifying one class over another, thereby affecting which rules get selected for the classifier. Originally, these weights were selected manually, which relied on human intuition and resulted in a tedious, reiterative process of selecting weights, building a model, and testing it on the fine-tuning data set in order to find the best classifier on the fine-tuning data set. The enhancement automates the process of accepting user-specified ranges for the weights on each class. Using this enhancement, all possible models were built and tested on the fine-tuning data set. Models that maximized certain metrics were retained.

The second enhancement was to implement an additional classifier, named the *Weighted Voting Classifier*. Unlike other classifiers that make a prediction using only the highest ranked rule, this classifier instead finds all rules in the classifier that match the antecedents, and combines these rule predictions into the final prediction. The *Weighted Voting Classifier* is built in a similar fashion to but applied differently from the original classifier described in Buczak et al. [20]. All rules in the classifier that match the data point are found, and each of these rules casts a vote for its consequent that is weighted by the product of the rule's confidence and the rule's fuzzy support on that data point. The class with the highest vote total is used as the prediction for the given data point. Both the original classifier and the *Weighted Voting Classifier* are used with different support metrics to build the full range of models, all of which are tested on the fine-tuning data set to find the best model. The results are then reported on previously unused testing data set.

### Performance Metrics

Four metrics commonly used to represent accuracy were used to assess the accuracy of the prediction:

- Positive Predictive Value (PPV): 
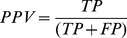
 which is the proportion of positive predictions that are outbreaks;
- Negative Predictive Value (NPV): 
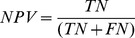
 which is the proportion of negative predictions that are non-outbreaks;
- Sensitivity: 
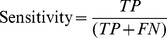
 which is the proportion of correctly predicted outbreaks (also called Probability of Detection);
- Specificity: 
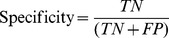
 which is the proportion of correctly predicted non-outbreaks; 1- Specificity is the False Alarm Rate;

where TP, TN, FP, and FN represent, respectively, True Positive, True Negative, False Positive, and False Negative. It should be kept in mind that, while these metrics are often used in medical diagnostic testing, the context here is one of predicting a future condition as opposed to detecting whether a condition is already present or not.

While these four metrics can be used to determine prediction accuracy, there is also a need to judge which of the created models is better in terms of how these results will be used in practice. For example, a high incidence prediction could lead to a public health department using its limited resources to deploy costly measures (e.g., mosquito spraying). One consideration is whether it is more important to use a model with a high PPV or a high Sensitivity. A high PPV indicates that, when the model predicts high incidence rate, high incidence is very likely to actually occur. When disease prevention and mitigation resources are limited, it is very important to have high PPV. Based on feedback from the local public health departments in Peru, there was a desire for a dengue prediction model with high PPV [20]. To reduce morbidity and mortality, public health departments also want to be able to mitigate any real outbreaks without being caught unprepared. A high Sensitivity indicates that the model predicts a high percentage of the outbreaks that actually occur. If PPV is high and Sensitivity is low, it means that when an outbreak is predicted, the probability is high that it will occur, but only a small percentage of actual outbreaks are predicted. In an attempt to illustrate these differences, the F-score [45] was introduced.

The F-score is a measure that considers both PPV and Sensitivity:
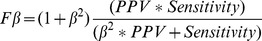



By varying the value of β, the resulting F-score will reflect the relative importance given to PPV and Sensitivity. When β equals one, PPV and Sensitivity are weighted equally. Assigning β a value less than one or greater than one gives more importance to PPV or Sensitivity, respectively. Therefore, F_0.5_ and F_3_ values give more importance to PPV and Sensitivity, respectively, and the performance of the models with the best F_0.5_ and F_3_ values will be presented in this paper.

### Training, Fine-tuning, and Testing Data Sets

The data were divided into three sets: training, fine-tuning, and testing. The *training data* were used to develop the models. In supervised learning, an automated classifier uses the training data set to learn about the nature of the problem. In the rule mining approach, all the rules with a support higher than the pre-defined support threshold and with a confidence higher than the pre-defined confidence threshold are extracted from the training data set and can potentially be used in the classifier. Classifiers are automatically built from subsets of these extracted rules using the training data. Candidate classifiers are scanned to pick the best classifier by minimizing a user-defined error on the *fine-tuning data* set (the error measures that we are maximizing are F_0.5_ and F_3_). Once the best two classifiers (optimizing the F_0.5_ and F_3_, to give more importance to PPV and Sensitivity, respectively) are selected, their performance is measured on the *testing data* set and reported as the classifier performance in terms of PPV, NPV, Sensitivity, and Specificity. The testing data set must be disjoint from training and fine-tuning data sets in order to provide a fair and objective indicator of the classifier performance on new/unseen data. In principle, the test error is considered to be an unbiased estimate of the true model error. As mentioned above, the test data used as model input are only those data that would actually have been available at the time the prediction was made.

## Results

The training data included 40 provinces and spanned January 2003–October 2010. The fine-tuning data included October 2009–October 2010 data for the same 40 provinces. The testing data spanned March 2011–December 2011 for 40 provinces. The results reported below are based only on the performance of the models in predicting the 2011 incidence data that were not used for model development. In addition to the results for 40 provinces with good data reporting, the results for all 81 provinces are also provided in order to determine how well the model can generalize to provinces that were never used in model development.

### Four Weeks Ahead Prediction Results

The method builds a large number of models (i.e., classifiers) that differ because of different rule selection parameters (i.e., criteria for selecting and excluding rules based on support, confidence, etc.) and different misclassification weights. The metrics (PPV, NPV, Sensitivity, Specificity, F_0.5_ and F_3_) for all classifiers are first computed on the fine-tuning data set. The two classifiers with the highest F_0.5_ (emphasis on PPV) and the highest F_3_ (emphasis on Sensitivity) on the fine-tuning data are selected as the final models computing predictions on the test data set.

The results obtained when optimizing for PPV and when optimizing for Sensitivity are shown in Tables 4 and 5, respectively. The most important results are the ones for the first test data set: this is the data set that is not used in training and fine-tuning the model, and that contains the same 40 provinces whose older data were used to develop the model.

**Table 4:** FARM prediction results for Philippines optimized for F_0.5_.

Data set	PPV	NPV	Sensitivity	Specificity	F_0.5_
**Fine-tuning set**	0.852	0.969	0.836	0.972	0.848
**Test set (2011 – 40 provinces)**	0.780	0.938	0.547	0.978	0.719
**Test set (2011 – all provinces)**	0.766	0.927	0.467	0.980	0.679
**Test set (2012 – 40 provinces)**	0.766	0.874	0.405	0.971	0.650
**Test set (2012 – all provinces)**	0.787	0.867	0.410	0.972	0.664

**Table 5:** FARM prediction results for Philippines optimized for F_3_.

Data set	PPV	NPV	Sensitivity	Specificity	F_3_
**Fine-tuning set**	0.656	0.990	0.952	0.904	0.911
**Test set (2011 – 40 provinces)**	0.778	0.948	0.627	0.974	0.639
**Test set (2011 – all provinces)**	0.748	0.938	0.555	0.973	0.570
**Test set (2012 – 40 provinces)**	0.733	0.884	0.467	0.960	0.484
**Test set (2012 – all) provinces**	0.762	0.877	0.463	0.964	0.482

For the model with the optimized PPV (Table 4), the test set PPV was 0.780 and the Sensitivity was 0.547. When all 81 provinces, including the ones with unreliable data reporting, were tested, both the PPV and Sensitivity showed small declines to 0.766 and 0.467, respectively. Thus, this model was able to generalize well even for provinces that were not used in training the model. Results obtained from the model optimized for Sensitivity on the test data from the 40 provinces in 2011 (Table 5) show a PPV and Sensitivity of 0.778 and 0.627, respectively. The PPV and Sensitivity for the 2011 data for all 81 provinces were 0.748 and 0.555, respectively.

Once the prediction models described above were developed and finalized, data were obtained for 2012. These new data were pre-processed and used as input to the models previously trained (i.e., no re-training was performed) to obtain predictions for 2012. The results show that the model optimized for PPV (Table 4), without any retraining, remains relatively robust: results are only slightly lower for 2012 than for 2011 data.

The model optimized for Sensitivity (Table 5) shows more variation from 2011 to 2012 than the model optimized for PPV: Specificity and PPV stay at about the same level, whereas NPV and Sensitivity are decreased. This variation is also shown as a drop in F_3_ values from 0.639 to 0.484. Overall, the models are relatively robust: their performance decreases gracefully when testing on data two years after the model training data, and when testing on data from provinces that were never used in training.

**Figure 5:**
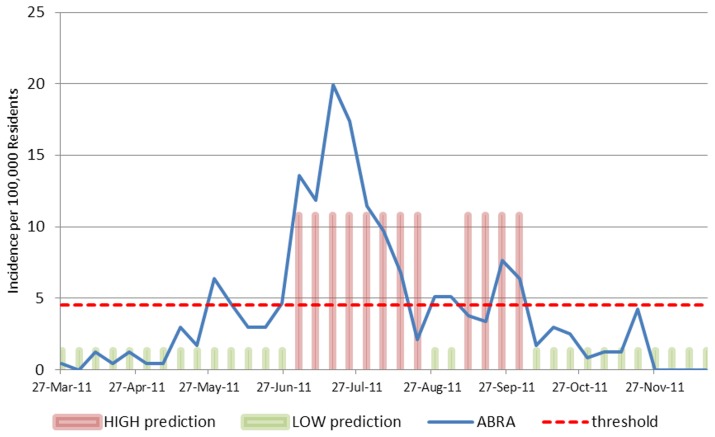
Incidence rate and predicted incidence rate for the province of Abra. Green bars correspond to prediction of LOW and red bars correspond to prediction of HIGH. When the incidence rate exceeds the threshold and a red bar is present, this corresponds to a TP; when the incidence rate is below the threshold and a green bar is present, this corresponds to a TN; when the incidence rate is above the threshold and a green bar is present, this corresponds to a FN.

Figure 5 shows the actual and predicted weekly incidence (4 week ahead prediction) for the province of Abra using the prediction model from Table 4. There are two missed weekly HIGH incidences near 27 May 2011 and 27 September 2011, but most of the predictions are correct.

**Figure 6:**
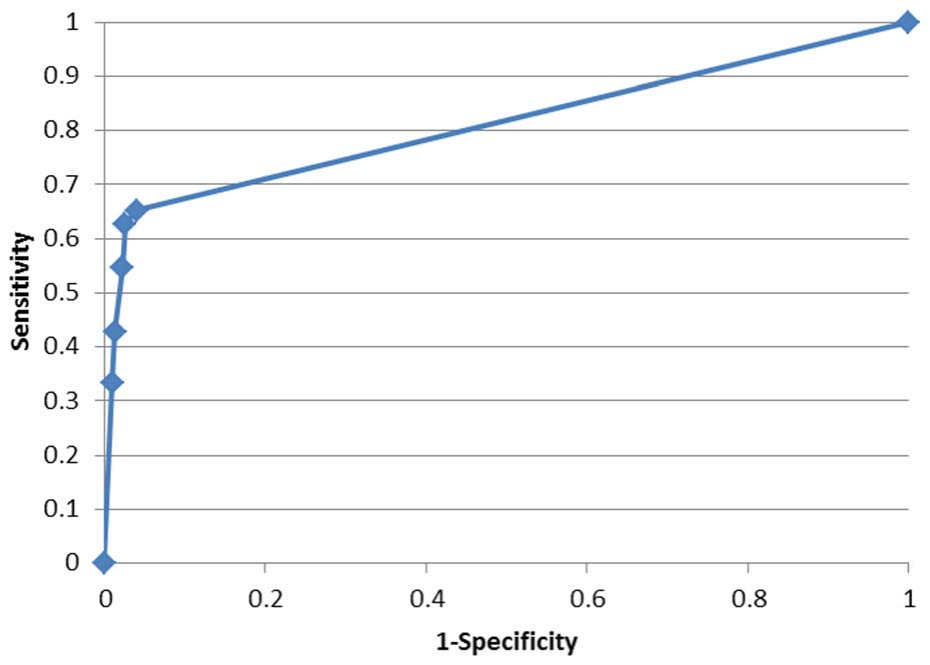
ROC curve for Philippines' predictions four weeks in advance.

**Figure 7:**
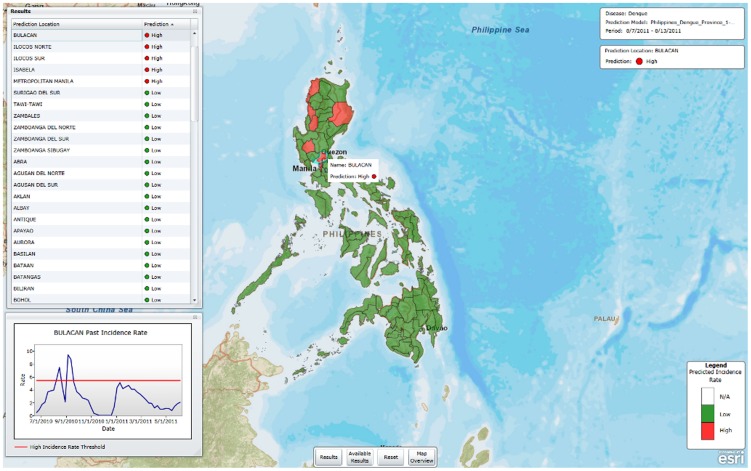
Four-week ahead prediction for the Philippines for the week 8/7–8/13/2011.

Figure 6 shows the Receiver Operating Characteristic (ROC) curve for the dengue prediction models developed by the method presented. Figure 7 shows a map with the model prediction for the week 8/7–8/13/2011 made using data that would actually have been available on 7/10/2011. For 12 provinces, the predictions are HIGH incidence (shown in red) and for the remaining provinces the predictions are LOW incidence (shown in green). This type of map could be useful for public health professionals who would then have four weeks in which to prepare and implement mitigation strategies for the provinces predicted to have HIGH incidence.

**Figure 8:**
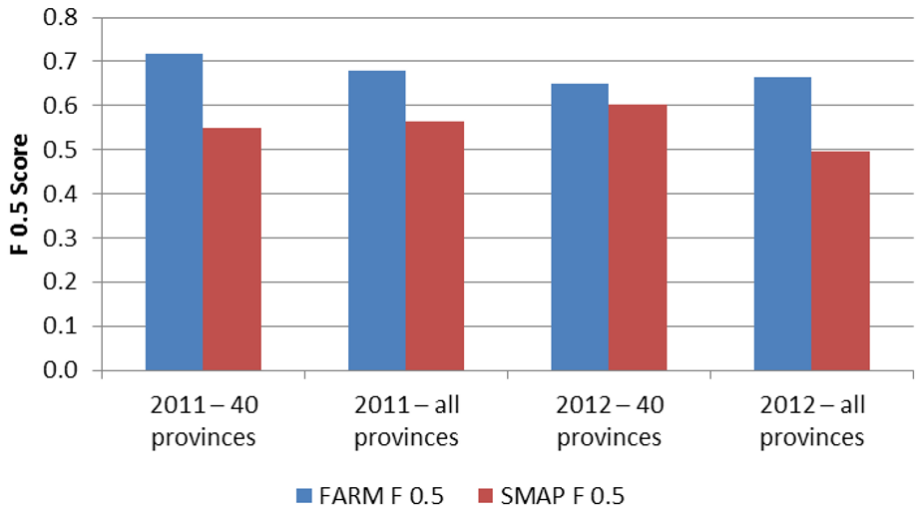
Comparison of F0.5 using four data sets for simple autoregression (SP) and the FARM method used in this paper.

**Figure 9:**
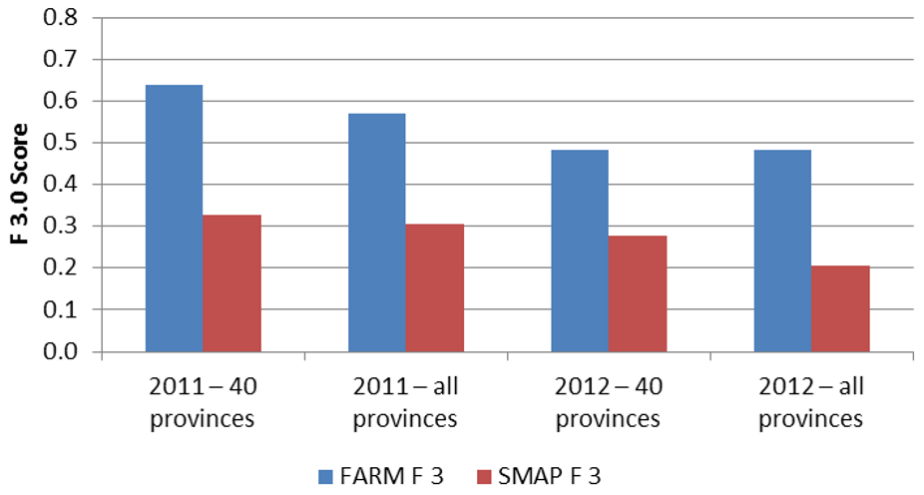
Comparison of F3.0 using four data sets for simple autoregression (SP) and the FARM method used in this paper.

For comparison of these results with another simpler method, predictions were also made using a seasonal moving average method that uses only the weekly incidence values from the previous five years for prediction:
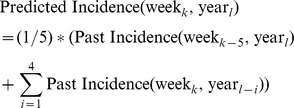



When making a prediction for week k of the current year, note that week k-5 represents the most recent data values available for making a prediction 4 weeks in advance (similar to what our method uses). This seasonal moving average prediction (SMAP) is the average of the week *k-5* dengue data from the current year and the week k dengue data from the four previous years. The results of the seasonal moving average prediction are shown in Table 6. Figures 8 and 9 show a comparison between the SMAP model and FARM models optimized for PPV and Sensitivity, respectively. The FARM model performs better in terms of Sensitivity, F_0.5_, and F_3_ on all data sets, and has a higher PPV on 2 out of 4 data sets. On the remaining two data sets, the higher PPV for the SMAP method is achieved at the expense for a very low Sensitivity and low F_0.5_ compared to the FARM method.

**Table 6:** Seasonal moving average prediction results for Philippines.

Data set	PPV	NPV	Sensitivity	Specificity	F_0.5_	F_3_
**Test set (2011 – 40 provinces)**	0.681	0.908	0.308	0.979	0.548	0.326
**Test set (2011 – all provinces)**	0.745	0.906	0.287	0.986	0.565	0.306
**Test set (2012 – 40 provinces)**	0.904	0.837	0.257	0.993	0.601	0.277
**Test set (2012 – all) provinces**	0.836	0.814	0.189	0.99	0.496	0.205

## Discussion

The work presented herein describes a method that uses novel data mining techniques to sift through large quantities of disparate data in order to find associations among these data. The method automatically selects those associations that meet certain pre-defined criteria, and uses these selections as rules for a prediction model. This procedure thus limits the number of rules and variables to those that are most important for the prediction. The resulting model is objective and reproducible. Past disease incidence data, along with environmental and socio-economic variables that have been shown in the literature to influence potential exposure to the virus, should provide enough information to allow a prediction of whether there is likely to be high incidence of dengue at a specific time and place in the future. This prediction model should not be confused with a detection model, as it is not designed to be used for early detection of a possible outbreak that has already begun and is not yet obvious because it is in a prodromal stage, etc. Although it may complement such detection, this method is instead designed to predict whether or not a high incidence of disease (such as that due to an outbreak) will be occurring several weeks in the future.

As long as there are strong associations between the predictor data and the information to be predicted (e.g., outbreak of a certain disease), the method should be able to automatically build a prediction model with reasonable accuracy. The accuracy of the model depends also on the quality of the data (i.e., the higher the quality, the more accurate the model). This is especially important for the epidemiological data that may have inconsistent reporting over many years. For example, the number of cases may seem to be increasing, but this could be because cases were under-reported previously, or because disease reporting was recently improved by the addition of funding or other resources. Therefore, special care should be taken to see if such discrepancies are present in the data used to develop and fine tune the model. The data mining techniques used in this method are general in the sense that they can use any data. Provided that data of reasonable quality are available, using this method to predict high/low disease incidence for other mosquito-borne diseases is expected to provide similar performance; for example, our preliminary unpublished results for malaria appear promising. The models developed are robust and able to predict with reasonable accuracy even for those Philippine provinces that were never used in model development.

Model input data were those that would actually be available on the date the prediction was made (e.g., if T-1 data were typically not available at T, then the most recently available were used). This was done to make the metrics representative of actual use and to avoid what would effectively be a retrospective prediction. It is also important to note that prediction accuracy was measured using data never before seen by the model. As emphasized in Lowe et al. [18], doing so provides a more rigorous and objective test of model performance. The novel method described herein showed good performance in predicting multi-week dengue incidence four to seven weeks in advance in Peru [20], and current results demonstrate that the model predicts weekly dengue incidence in Philippines provinces four weeks in advance with high accuracy. Such advance notification could provide valuable time for public health professionals and others to prepare for and employ disease mitigation strategies, thereby reducing morbidity and mortality. Because the effectiveness of such mitigation could be expected to increase as advances in disease prevention become available for local public health departments, this ability of predictive modeling to gain time for mitigation and consequence management planning should become more valuable.
